# Resveratrol exhibits neuroprotection against paraquat-induced PC12 cells via heme oxygenase 1 upregulation by decreasing MiR-136-5p expression

**DOI:** 10.1080/21655979.2022.2045764

**Published:** 2022-03-02

**Authors:** Li Zhang, Min-Na Dong, Jun Deng, Chun-Hai Zhang, Ming-Wei Liu

**Affiliations:** a Department of Neurology, Yan-an Hospital of Kunming City, Kunming, China; b Department of Emergency Medicine, The First Affiliated Hospital of Kunming Medical University, Kunming, China

**Keywords:** Oxidative stress, miR-136-5p, nuclear factor-erythroid factor 2-related factor 2, paraquat, PC12 cells, heme oxygenase 1

## Abstract

Resveratrol (Res) is a flavonoid with an antioxidant effect and has been utilized to treat oxidative stress-related illnesses; however, its mechanism remains ambiguous. This research aims to explore whether Res inhibits miR-136-5p expression, increases heme oxygenase 1 (HMOX1) expression, and mitigates oxidative stress and PC12 cell apoptosis triggered by paraquat (PQ). Results showed that PQ dose-dependently increased the expression of miR-136-5p, the apoptosis of PC12 cells, the activities of reactive oxygen species (ROS), and the levels of lactate dehydrogenase (LDH), malondialdehyde (MDA), caspase-3, and pro-apoptotic protein Bax. In addition, PQ reduced the expression of anti-apoptotic protein Bcl-2, HMOX1 mRNA and protein, and nuclear factor-erythroid factor 2-related factor 2 (Nrf2) protein and the activity of superoxide dismutase 1 (SOD1) and PC12 cells. After the PQ-treated PC12 cells were administered with different Res concentrations for 24 h, the miR-136-5p expression was dose-dependently decreased. An increase was observed in the activity and survival rate of PC12 cells, the protein and mRNA levels of HMOX1 and Nrf2, and the content of anti-apoptotic protein B-cell lymphoma/leukemia gene-2 (Bcl-2). By contrast, the activities of ROS, LDH, and MDA and the apoptosis of PC12 cells decreased. These findings illustrated that Res could reduce the oxidative stress and apoptosis triggered by PQ and enhance the activity and survival rate of PC12 cells. The underlying mechanism might be correlated with the reduced miR-136-5p expression and the elevated activity of the HMOX1/Nrf2 pathway.

## Graphical abstract

**Figure:**
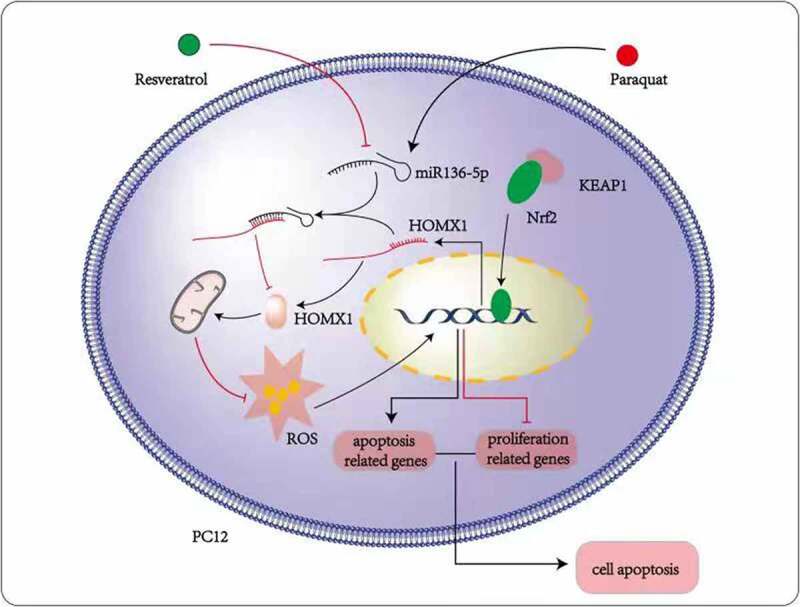

## Introduction

Paraquat (PQ) is widely used for weeding in wheat fields, and exposure to this substance remarkably increases the risk of Parkinson’s disease (PD) in agricultural workers. Compared with that in non-exposed individuals, PD risk is six times higher in a patient exposed to PQ for 20 years. A dose–effect relationship is observed in PQ application [1]. After acute poisoning, PQ is mainly concentrated in the hypothalamus and other parts that are not protected by the blood–brain barrier. This substance changes norepinephrine release, inhibits hypothalamic function, and induces weight loss. Hydrocephalus and hemorrhage are noted in severe cases [2]. Grant and Hughes et al. conducted an autopsy study on patients who died due to PQ poisoning. The results showed diffusible brain edema and deep white matter lesions mainly located within the third ventricle and the lateral ventricle, particularly in the bilateral brain parenchyma within the subarachnoid space [3,4]. When administered systemically to mice, PQ causes various neuropathological characteristics that are indicative of PD [5]. Current treatments based on the assumed mechanism of this agent failed to produce successful results.

Oxidative stress is a major factor of cell damage in many neurological disorders, such as cerebral ischemia–reperfusion injury, Alzheimer’s disease, epilepsy, and PD, and can lead to nerve cell death by inducing apoptosis [6,7]. When 1-methyl-4 phenyl-1,2,3,6-tetrahydropyridine (MPTP) enters the brain, it becomes a neurotoxin that transforms into ionic MPP+ and is discharged extracellularly through monoamine oxidase (MAO) A and B activity in glial cells [8].When MPP+ enters the mitochondria of dopaminergic neurons through the dopaminergic transporter (DAT), it blocks the action of mitochondrial complex I, interrupts ATP production, and induces the aberrant production of reactive oxygen species (ROS), thus eventually resulting in selective dopaminergic neuronal degeneration and death [9]. PQ is commonly utilized as a nonselective herbicide and has a chemical structure similar to that of MPP+ (1-methy1-4-pehny1-pyridine, MPP+) and its active metabolite1-methyl-4-phenyl-1,2,3,6-tetrahydropyridine (MPTP) [6]. This similarity in structure might be one of the underlying factors of PD pathogenesis. PQ can selectively destroy dopaminergic neurons located within the substantia nigra striatum of experimental animals [7]. Hence, its neurotoxic effects have begun to attract research attention.

Nuclear factor-erythroid 2-related factor 2 (NRF2) is an important oxidative stress regulator protein, and its pathway is activated by the disrupted interaction between NRF2 and Keap1. As an important defense mechanism against oxidative stress, managing the Keap1/NRF2/ARE pathway may provide beneficial effects on chronic diseases such as cardiovascular diseases, diabetes, atherosclerosis, cancer, and neurodegenerative diseases [10–12]. Oxidative stress dissociates the interaction between Keap1 and NRF2, with the latter translocating into the nucleus to heterodimerize with musculoaponeurotic fibrosarcoma oncogene homolog and interact with antioxidant response elements. The expression of downstream target genes such as HMOX1 and NQO1 is then induced to protect the cells from oxidative damage [11]. HMOX1 catalyzes heme to release free irons to form biliverdin, which further metabolizes to carbon monoxide (CO) and bilirubin with antioxidative effect [12]. Therefore, we speculate that the NRF2/HMOX1 pathway may be involved in PQ-induced PC12 cell damage.

MicroRNAs (miRNAs) are small non-coding RNAs that control the expression of complementary target messenger RNAs [13]. In vertebrates, miRNA expression is substantially higher in the brain than in other organs. MiRNAs are expressed during embryonic and mature stages and participate in various physiological and pathological processes that determine cell fate. Understanding their contribution to the development of the neurological system and the onset of different disorders is necessary. MiRNAs perform a crucial function in cell proliferation, differentiation, and apoptosis and are strongly associated with the onset and progression of diseases, such as epilepsy and cerebral infarction. These macromolecules have an essential role in epilepsy, PD, and cerebral infarction [13,14]; however, their mechanism is unclear.

Resveratrol is a natural polyphenolic compound that is mainly found in grapes, peanuts, and *Polygonum cuspidatum* and is one of the active antitoxin components of stilbene plant. This substance exhibits a neuroprotective effect against neurological diseases, including PD stroke, epilepsy, pain, and Alzheimer’s disease [15–17]. Its functions generally include anti-oxidation, anti-inflammatory, mitochondrial protection, and/or autophagy induction [18]. However, its specific molecular mechanisms and roles in different diseases have not yet been clarified.

MiR-136-5p is overexpressed in brain tissues and regulates the inflammation and oxidative stress caused by ischemia–reperfusion injury via regulating Rho-associated coiled-coil-containing protein kinase [19]. HMOX1 is an important antioxidant protein in the body and can effectively reduce its oxidative stress response [20]. HMOX1 was identified as a possible regulatory target of miR-136-5p by Targetscan. Resveratrol offers remarkable protection from severe oxidative stress and inflammation and ameliorates the general well-being of mice against the toxic outcome of PQ [21]. In this experiment, we hypothesized that HMOX1 is a regulatory target of miR-136-5p. In addition, resveratrol increases HMOX1 expression and reduces PQ-induced oxidative stress and apoptosis in PC12 cells by downregulating miR-136-5p expression.

## Materials and methods

### Cell culture

Murine adrenal pheochromocytoma cell line PC12 was provided by the Shanghai Chinese Academy of Sciences Cell Bank (Shanghai, China). The PC12 cells were removed from the cell bank, quickly thawed, transferred to a centrifuge tube on an ultra-clean table, and added with an appropriate amount of prepared fresh medium. After centrifugation, the sediment was removed, and the culture was added with fresh Dulbecco’s modified eagle medium comprising 10% fetal bovine serum, 100 μg/mL streptomycin, and 100 U/mL penicillin. The sample was routinely placed in a humidified incubator at 37°C, 5% CO_2_ concentration, and 95% air. The cells in log growth phase were used for the experiment. Resveratrol dose was set as 25–75 μmol/L [22]. The cells were subsequently classified into normal control, PQ induction, PQ + resveratrol (25 μmol/L), PQ + resveratrol (50 μmol/L), and PQ + resveratrol (75 μmol/L) groups to detect the impact of resveratrol on PQ-induced cell damage. The PC12 cells were added with different PQ concentrations (200–1000 μmol/L [23,24] and cultured for 24 h to determine various indicators.

### Cell transfection

An appropriate amount of PC12 cells in the log growth stage were seeded onto a six-well plate. After attaching to the wall overnight and growing to 60% confluence, the cells were transfected with miR-136-5p inhibitor, miR-136-5p mimic, inhibitor control, and mimic control using Lipofectamine 2000 transfection reagent in accordance with the operating instructions. The concentration used for transfection was 50 nmol/L. The medium was replaced with a fresh one every after 6 h, and the culture was permitted to continue for another 24 h. The cells were acquired for subsequent analyses.

### Detection of cell proliferation utilizing the 3-(4,5-dimethylthiazol-2-yl)-2,5-diphenyltetrazolium bromide (MTT) method

In brief, 5 × 10^7^/L PC12 cells in log growth phase were placed in a 96-well plate at a concentration of 100 μL cells per well and then placed in a 5% CO_2_ incubator to facilitate the attachment of the cells to the wall. Before the medicine was added the next day, the old medium was removed. Five multiple wells were set in each group. In the experimental group, 100 μL per well was added with various PQ(200, 400, 800, and 1000 μmol/L) or PQ (800 μmol/L) + resveratrol (25, 50, and 75 μM) concentrations. The culture was incubated in a 96-well plate for 24 h. Each well was then added with 20 μL of 5 g/L MTT solution [25], followed by another incubation period of 4 h. In each well, 150 μL of DMSO was introduced once the supernatant had been removed. The sample was shaken to allow crystal dissolution. The absorbance (A) of the cells was detected at a wavelength of 490 nm. Cell survival rate (%) = (experimental group A-blank group A)/(negative control group A-blank group A) × 100%. Each experiment was conducted at least three times.

### Detection of cell viability using cell counting kit-8 (CCK-8)

Cells in the logarithmic growth phase were placed in a 96-well plate with a cell concentration of 5 × 10^7^/L at 100 μL of cells per well and then placed in a 5% CO_2_ incubator to make the cells adherent. The old medium was removed before the medicine was added on the next day. Five multiple wells were set in each group, and each well of the experimental group with a volume of 100 μL was added with different concentrations of PQ (200, 400, 800, 1000 μmol/L) or PQ (800 μmol/L) + resveratrol (25, 50, and 75 μM) and cultured for 24 h. The blank group had no cells and only medium, and the control group was only added with the same medium. After the drug was administered, incubation was continued in a 96-well plate for 24 h [26], and 20 μL of CCK-8 solution was added in each well. The plate was incubated again for 1 h at 37°C to terminate the culture. The blank well was set to zero, and the plate was oscillated on the microplate reader for 3 min. The OD value of each well was detected at 450 nm wavelength, and cell viability was recorded and calculated.

### Detection of apoptosis

PC12 cells were placed on a six-well culture plate, added with varying amounts of PQ (200, 400, 800, 1000 μmol/L) or PQ (800 μmol/L) + resveratrol (25, 50, and 75 μM), and incubated for 24 h. The cells were the collected, washed three times using PBS, and centrifuged at 1200 r/min for 5 m. In brief, 200 μL of 1× binding buffer was added to form cell suspension. The mixture was added with 5 μL of annexin V-EGFP and 5 μL of propidium iodide [27] and reacted for 15 min at ambient temperature in darkness. A flow cytometer was used at the emission wavelength of 530 nm and excitation wavelength of 488 nm. The FITC channel detects the annexin V-EGFP’s green fluorescence, and the PI channel detects the PI’s red fluorescence. Normal cells without the apoptosis induction treatment were used as control for fluorescence compensation adjustment to reduce the spectral overlap and establish the location of the cross gate.

### Detection of ROS in cells

For PC12 cell liquid preparation, cells in the logarithmic growth phase were inoculated on a six-well culture plate at 2 × 10^5^/mL and added with different concentrations of PQ (200, 400, 800, 1000 μmol/L) or PQ (800 μmol/mL) + resveratrol (25, 50, and 75 μM). Afterward, the cells were collected, suspended in a serum-free culture medium containing10 μmol/L H2DCFDA [28], and incubated for 20 min in a 37°C cell incubator. The container was mixed upside down every 3–5 min to allow complete contact between the cells and the H2DCFDA fluorescent probe. The cells were washed once using serum-free cell culture solution and then twice with PBS solution to completely eliminate the H2DCFD that has not entered the cells. Each treatment was performed at least three times.

### Detection of SOD and LDH activity and MDA content

PC12 cells were plated on six-well culture plates and treated with various doses of PQ (200, 400, 800, and 1000 μmol/L) or PQ (800 μmol/L) + resveratrol (25, 50, and 75 μM). After culturing for 24 h, collect the cells, homogenize the cells of each group and place them on ice for lysis, centrifuge to collect the supernatant, use the SOD activity detection kit for SOD activity detection, LDH activity detection kit for LDH activity detection, and MDA content detection kit for the determination of MDA content.

### Detection of cell apoptosis and morphology using the AO/EB method

PC12 cells in logarithmic growth phase were evenly distributed and inoculated in a six-well plate at 3 mL per well with a density of 5 × 10^7^/L. The cells were cultivated in a constant temperature incubator to make them adhere to the wall. Different concentrations of PQ (200, 400, 800, and 1000 μmol/L) or PQ (800 μmol/L) + resveratrol (25, 50, 75 μM) were added. The cells were incubated for 24 h, collected, and washed twice by adding 1 mL of PBS solution into each well. In brief, 20 μL of working solution with 1:1 EB and AO ratio was added to each well and left for 5 minutes at ambient temperature [29]. Observation and imaging were performed with a fluorescent inverted microscope.

### Protein extraction and Western blot

PC12 cells were seeded in six-well culture plates, added with various doses of PQ (200, 400, 800, 1000 μmol/L) or PQ (800 μmol/L) + resveratrol (25, 50, 75 μM), and incubated for 24 h. Afterward, the cells were collected, washed three times with PBS, and lysed with RIPA lysis buffer containing protease and phosphatase inhibitors. The BCA kit was used to determine protein content and prepare samples [30]. For SDS-PAGE gel preparation, a loading program of 30 min of 70 V and subsequent 50 min of 140 V electrophoresis was conducted. After electrophoresis, NC membrane 18 V 350 mA semi-wet transfer membrane was used for 20 min, and 5% PBS milk powder blocking was employed for 30 min. The membrane was cut after being washed with PBST three times for 5 min each. The primary antibody (β-actin, HMOX1, Bax, Nrf2, Bcl-2, caspase-3 diluted 1:1 000) was incubated at 4°C overnight and then collected. Secondary goat anti-mouse IgG-HRP antibody (1:2000) or goat anti-rabbit IgG-horseradish peroxidase (HRP) antibody (1:2000) was acquired from Santa Cruz Biotechnology (Santa Cruz, CA, USA) and diluted in 5% (w/v) dry nonfat milk in TBST at ambient temperature for 1 h. All outcomes were normalized to β-actin levels by lane. All Western blots were quantified using densitometry.

### Real-time quantitative PCR (RT-qPCR)

PC12 cells were seeded in six-well culture plates, added with various doses of PQ (200, 400, 800, 1000 μmol/L) or PQ (800 μmol/L) + resveratrol (25, 50, 75 μM), and cultivated for 24 h. The cells were collected by centrifuging at 2000 g/min. After washing with PBS, the TRIzol method was utilized to extract total RNA, and reverse-transcription of mRNA into cDNA was conducted by M-MLV reverse transcriptase in accordance with PrimeScript TM RT reagent Kit instructions. RT-qPCR was used to evaluate the expression level of SOD1 mRNA in cells. The following describes the reaction system: DEPC water 6 μL, DNA template 2 μL, PCR primers 2 μL, SYBR Premix Ex TaqTMII10 μL, and total reaction system 20 μL. The primer sequences are shown in Table 1. After 10 min pre-incubation at 95°C, PCR was performed using the following program: 35 cycles of denaturation at 95°C for 15s, annealing at 60°C for 5s, and elongation at 72°C for 12s. 2^−ΔΔCt^ method was utilized to assess the mRNA expression levels of target genes [31]. Cycle threshold (Ct) was measured, and the number of miR-136-5p with respect to U6 RNA was calculated using the mathematical formula of 2^−(CtmiRNA2CtU6RNA)^.

**Table 1.** Primer sequences for RT-PCR analysis

Primer name	Sequence (5’-3’)	Product
miR-136-5p	F-5’-ACTCCATTTGTTTTGATGATGGAA-3’	328
	R-5’- GCTGTCAACGATACGCTACGTAAC-3’	
U6	F-5’- CCTGCTTCGGCAGCACA −3’	241
	R-5’-AACGCTTCACGAATTTGCGT-3’	
HMOX1mRNA	F-5’- CGAGGTGGGAGGTACTCATC −3’	226
	R-5’- TGAGTGTGAGGACCCATCG-3’	
β-actin	F-5’ -CCAACCGTGAAAAGATGACC −3’	259
	R-5’- ACCAGAGGCATACAGGGACA-3’	

### Double luciferase reporter gene detection experiment

Target gene prediction software PicTar (http://pictar.m dc-berlin.de/), TargetScan (http://www.targetscan.org/), and miR-Base (http://www.mirbase.org/) were utilized to predict whether miR-136-5p targets HMOX1. Following a previous method [7], mutant (mut)-HMOX1 and wild-type (wt)-HMOX1 luciferase gene vectors were generated, combined with miR-136-5p inhibitor, miR-136-5p mimic, inhibitor control, and mimic control, and co-transfected into PC12 cells induced by PQ (800 μmol/L) and counted as Mut + negative control, Wt + negative control, Mut + miR-136-5p, and Wt+miR-136-5p mimic, respectively. The dual-luciferase reporter gene kit was utilized to identify the presence of dual-luciferase activity after the cells were incubated at 37°C and 5% CO_2_ for 48 h. RT-PCR and Western blot were utilized to identify the level of HMOX1 in PC12 cells that overexpressed miR-136-5p. The ratio of the luminescence intensity of firefly luminescence intensity to the sea cucumber luciferase represents the strength of the bond between miR-136-5p and HMOX1.

### Immunofluorescence

The PC12 cells were plated onto 24-well plates to determine their Ki67 expression [32]. Incubation with Ki67 antibodies was performed after the cells were subjected to a series of pretreatments and treatments. The cells were stained with DAPI, treated with a fluorescent secondary antibody, and imaged with a fluorescence microscope.

### Statistical analysis

SPSS Statistics (version: 17.0) was utilized to conduct the statistical analyses. The measurement data are all normally distributed (P > 0.05) after normal inspection and articulated as mean ± standard deviation. After the homogeneity of variance test, the mean square deviation was uniform (P > 0.05), and one-way ANOVA was used to perform the SNK test and LSD-t analysis to make paired comparisons between groups. P < 0.05, p < 0.01, and p < 0.001 were considered as significant.

## Results

### PQ increased the oxidative stress, LDH activity, and apoptosis but reduced the viability of PC12 cells

**Figure 1.**
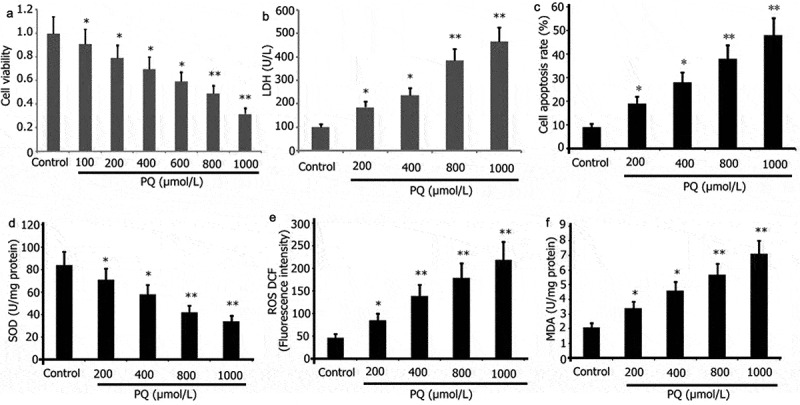
Impact of PQ on the cell viability, LDH activity, apoptosis, and oxidative stress of PC12 cells. After 24 h of incubation with PQ at increasing doses (200, 400, 800, and 1000 umol/L), cell viability was ascertained by CCK-8, and cell apoptosis was ascertained by flow cytometry. SOD and LDH activities and MDA levels were determined. The results from three separate experiments are expressed as mean ± SD. *P < 0.05,**P < 0.01versus the control group.

PQ can easily lead to oxidative stress in brain tissue, nerve cell apoptosis, and aggravate brain damage. PC12 cells were incubated with various doses of PQ (200, 400, 800, and 1000 μmol/L) for 24 h to observe the impact of PQ. CCK-8 assay was used to examine cell viability, and flow cytometry was utilized to assess the level of apoptosis in PC12 cells. ROS/MDA levels and SOD/LDH activities were measured. As illustrated in Figure 1, PQ attenuated PC12 cell viability (Figure 1(a–b)) but increased PC12 cell oxidative stress, LDH activity (Figure 1(d–f)), and apoptosis (Figure 1(c)).

### PQ increased the expression of pro-apoptotic protein and miR-136-5p and blocked the activity of Nrf2/HMOX1 signaling pathway in PC12 cells

**Figure 2.**
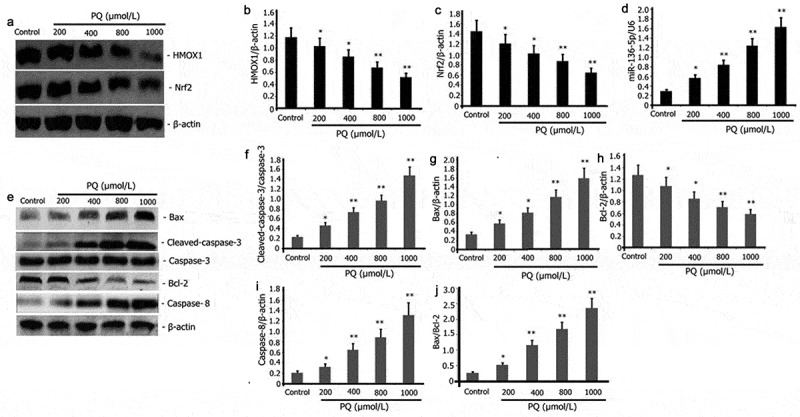
Impact of PQ on apoptotic protein, miR-136-5p expression, and Nrf2/HMOX1 signaling pathway activity in PC12 cells. After 24 h of incubation with increasing PQ concentrations (200, 400, 800, and 1000 umol/L), the expression of Bax, caspase-8, caspase-3, Bcl-2, Nrf2, and HMOX1 was determined by Western blot. RT-PCR was employed to estimate the expression of miR-136-5p.The results from three separate experiments are expressed as mean ± SD. *P < 0.05,**P < 0.01versus the control group.

MiR-136-5p and Nrf2/HMOX1 signaling pathway regulate cellular oxidative stress and apoptosis [33]. PC12 cells were incubated for 24 h with increasing doses of PQ (200, 400, 800, and 1000 μmol/L) to evaluate the impact of PQ on the expression levels of pro-apoptotic protein and miR-136-5p and the activity of Nrf2/ HMOX1 signaling pathway in PC12 cells. PT-PCR was performed to estimate the levels of miR-136-5p expression, and Western blot was utilized to determine the expression levels of caspase-3, Bax, Bcl-2, caspase-8, Nrf2, and HMOX1. As illustrated in Figure 2, PQ attenuated Nrf2 and HMOX1 expression (Figure 2(a–b)) and increased miR-136-5p (Figure 2(c)), caspase-3, caspase-8, Bax/Bcl-2 and pro-apoptotic protein Bax (Figure 2(e–j)) expression in PC12 cells.

### Resveratrol increased PQ-induced PC12 cell viability, LDH activity, and apoptosis

**Figure 3.**
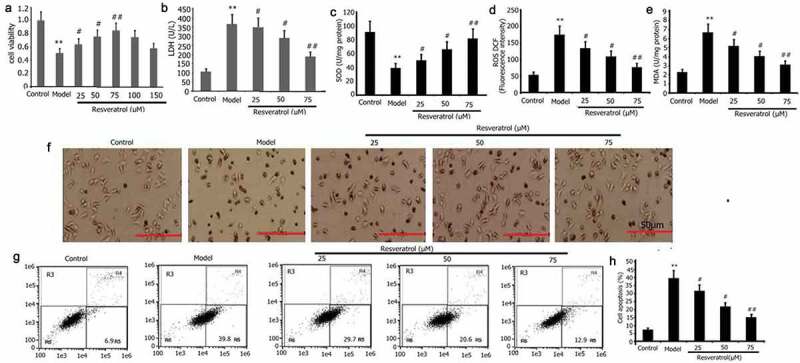
Impact of resveratrol on the viability, oxidative stress, LDH activity, apoptosis, and morphology of PC12 cells treated with PQ. PC12 cells were treated with PQ (800 umol/L) and different resveratrol doses (25, 50, and 75 M) for 24 h. Cell viability was determined by CCK-8, and LDH activity was measured following the manufacturer’s instructions. Morphology was assessed by AO/EB analysis, and SOD activity and ROS and MDA levels were evaluated. Cell apoptosis was determined by flow cytometry. The results from three separate experiments are expressed as mean ± SD. *P < 0.05, **P < 0.01versusthe control group. ^#^P < 0.05, ^##^P < 0.01versus the model group.

Resveratrol has anti-apoptotic effects and protects against cell damage from various causes. To analyze the impact of resveratrol on PQ-induced PC12 cell viability and cell viability, the activity of LDH, and apoptosis, the PC12 cells were subjected to incubation for 24 h with PQ (800 μmol/L) and various resveratrol concentrations (25, 50, and 75 μM). CCK-8 was utilized to assess the viability of PC12 cells. LDH activity was measured according to manufacturer’s instructions, and flow cytometry was utilized to measure apoptosis. As illustrated in Figure 3. Resveratrol enhanced PQ-induced PC12 cell viability (Figure 3(a-b)) and decreased PQ-induced PC12 cell the activity of LDH and apoptosis (Figure 3(g-h)).

### Resveratrol decreased PQ-induced PC12 cells oxidative stress, and repair PC12 cell damage induced by PQ

Resveratrol inhibits PQ-induced oxidative stress and reduces paraquat-induced tissue cell damage. To analyze effect of Resveratrol on PQ-induced PC12 cells oxidative stress and cell damage induced by PQ [34]. The PC12 cells were subjected to incubation for 24 h with PQ (800 μmol/L) and different doses of resveratrol (25 μM, 50 μM, and 75 μM) to investigate how resveratrol affects PQ-induced oxidative stress in PC12 cells. Furthermore, the levels of MDA and ROS, as well as the activity of SOD, were assessed. As shown in Figure 3, the levels of MDA and ROS were greatly reduced, while the SOD activity was considerably increased after resveratrol administration for 24 h. In the control group, the PC12 cells are evenly distributed, and the cell bodies emit irregular protrusions. The cells in the model group have a large number of floating cells and vacuoles. The cells are aggregated, the refractive index is increased, and their bodies have atrophied and become rounded and dark in color (Figure 3(f)). After the cells in the model group are treated with resveratrol, their morphology improved significantly, the number of adherent cells increased significantly, the number of aggregated and atrophied cells decreased significantly, and the refractive index of cells decreased (Figure 3(f)).

### Resveratrol attenuated the expression of miR-136-5p and pro-apoptotic protein and increased the activity of the Nrf2/ HMOX1 signaling pathway in PC12 cells

**Figure 4.**
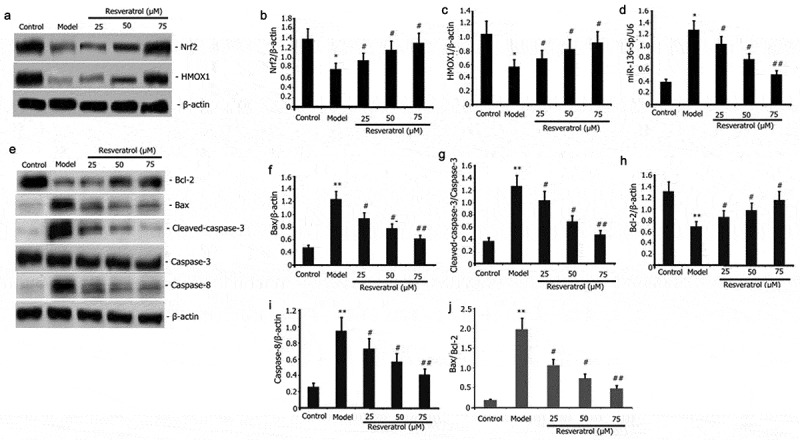
Impact of resveratrol on miR-136-5p expression, apoptotic protein, miR-136-5p expression, and Nrf2/HMOX1 signaling pathway activity in PQ-induced PC12 cells. PC12 cells were treated with PQ (800 umol/L) and resveratrol at varying doses (25, 50, and 75 M) for 24 h. MiR-136-5p expression was estimated by RT-qPCR. Western blot analysis was used to determine the expression of Bax, caspase-8, caspase-3, Bcl-2, Nrf2, and HMOX1 protein. The results from three separate experiments are expressed as mean ± SD. *P < 0.05versus the control group. ^#^P < 0.05, ^##^P < 0.01versus the model group.

Resveratrol regulates oxidative stress-induced apoptosis by regulating oxidative stress pathway, miR-136-5p and pro-apoptotic protein. PC12 cells were incubated with PQ (800 μmol/L) and various resveratrol doses (25, 50, and 75 μM) for 24 h to further evaluate how resveratrol affects the expression of pro-apoptotic protein and miR-136-5p and the activity of Nrf2/ HMOX1 signaling pathway in PQ-induced PC12 cells. RT-qPCR was utilized to measure the miR-136-5p expression, and Western blot was employed to determine the protein expression of Nrf2, HMOX1, caspase-3, Bcl-2, Bax, and caspase-8. As illustrated in Figure 4, the expression of Nrf2 and HMOX1 protein was considerably elevated (Figure 4(a–c)), whereas that of pro-apoptotic protein Bax, caspase-8, Bax/Bcl-2 and caspase-3, and miR-136-5p was substantially reduced (Figure 4(d–j)). Anti-apoptotic protein Bcl-2 expression was increased after resveratrol administration for 24 h.

### HMOX1 is a regulatory target of miR-136-5p

**Figure 5.**
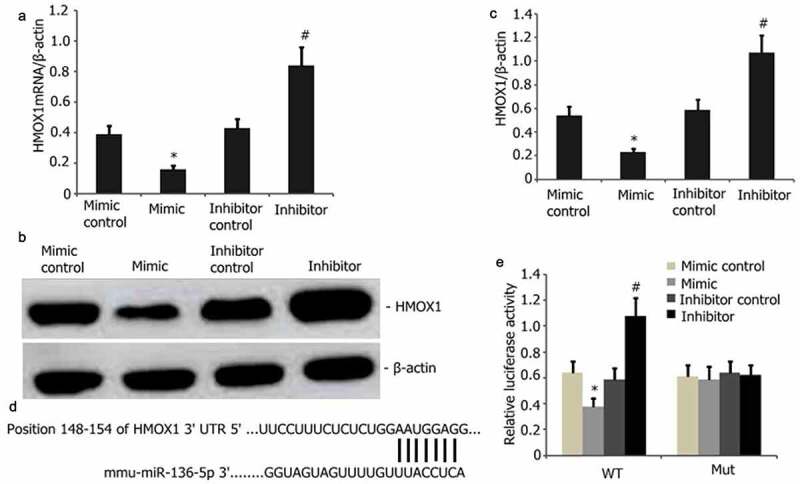
HMOX1 is a downstream miR-136-5p target. A–B: HMOX1 was reduced in PQ-induced PC12 cells with overexpressed miR-136-5p compared with that in the cells with empty vector control (P < 0.05). HMOX1 expression was substantially elevated in the cells with miR-136-5p down-modulation. C–D: HMOX1 mRNA levels decreased after miR-136-5p overexpression in PQ-induced PC12 cells (P < 0.05). Elevated levels following the up-modulation of miR-136-5p (P < 0.05). E: miR-136-5p bound to the 3ʹ-UTR regions of the HMOX1.In mutant HMOX1, the binding process was disrupted. F: Dual-luciferase reporter assay revealed that miR-136-5p mimic bound to the 3ʹ-UTR region of wild-type HMOX1 but not of HMOX1 mutants (P < 0.05).

HMOX1 and miR-136-5p can regulate PQ-induced oxidative stress and apoptosis. The potential targets of miR-136-5p were predicted by TargetScan and then utilized to examine how miR-136-5p facilitates the PQ-induced apoptosis of PC12 cells and clarify the mutual regulatory relationship between HMOX1 and miR-136-5p. HMOX1 was discovered to be a critical modulator of PQ-reduced PC12 cells. The levels of HMOX1 protein and mRNA were considerably attenuated after miR-136-5p overexpression (Figure 5(a–b,d–e)). Mutant and wild-type HMOX1were generated and used in a dual-luciferase reporter test to ascertain the interplay between miR-136-5p and HMOX1 (Figure 5(c,f)). As predicted, miR-136-5p bound to the wild-type HMOX1 instead of the mutants (Figure 5(d)).

### Impact of resveratrol plus miR-136-5p mimic on the Nrf2/ HMOX1 signaling pathway activity and apoptotic protein in PQ-induced PC12 cells

**Figure 6.**
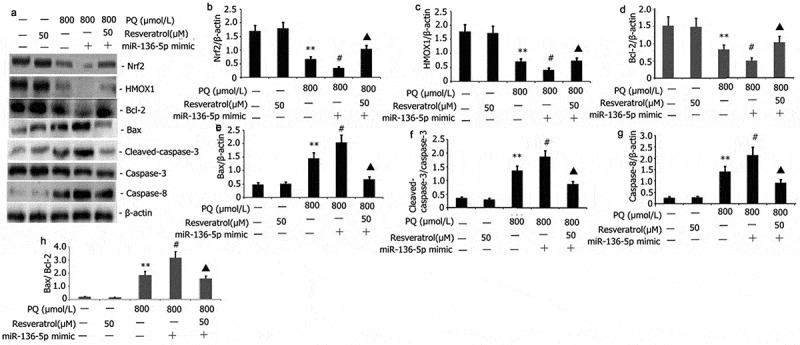
Impact of resveratrol plus miR-136-5p mimic on Nrf2/HMOX1 signaling pathway activity and apoptotic protein expression in PQ-induced PC12 cells. PQ (800 μmol/L) and different resveratrol doses (50 μM) were added to the PC12 cells before being transfected for 24 h using miR-136-5p mimic. Western blot was employed to examine the protein expression of Nrf2, HMOX1, Bax, caspase-8, and caspase-3. The findings are expressed as mean ± SEM. **P < 0.01 versus the control group and only resveratrol group. ^#^P < 0.05 versus the PQ group. ^▲^P < 0.05 versus the miR-136-5p mimic + PQ group.

PC12 cells were transfected with miR-136-5p mimic in the presence of PQ (800 μmol/L) and resveratrol (50 μM) for 24 to further study the effect of resveratrol plus miR-136-5p mimic on the Nrf2/HMOX1 signaling pathway activity and apoptotic protein in PQ-induced PC12 cells. Western blot was utilized to determine the levels of protein expression of Nrf2, HMOX1, Bax, caspase-8, and caspase-3. As shown in Figure 6, miR-136-5p mimic down-regulated the expression of Nrf2 and HMOX1 and up-regulated the protein expression of caspase-8, Bax, Bax/Bcl-2 and caspase-3 (Figures 6(a–g)). Resveratrol increased the expression of miR-136-5p mimic and Nrf2 and HMOX1 protein expression and attenuated the protein expression of Bax, Bax/Bcl-2, caspase-3, and caspase-8 in PQ-induced PC12 cells (Figures 6(a–g)).

### Impacts of resveratrol plus miR-136-5p mimic on the oxidative stress and morphology of PQ-induced PC12 cells

**Figure 7.**
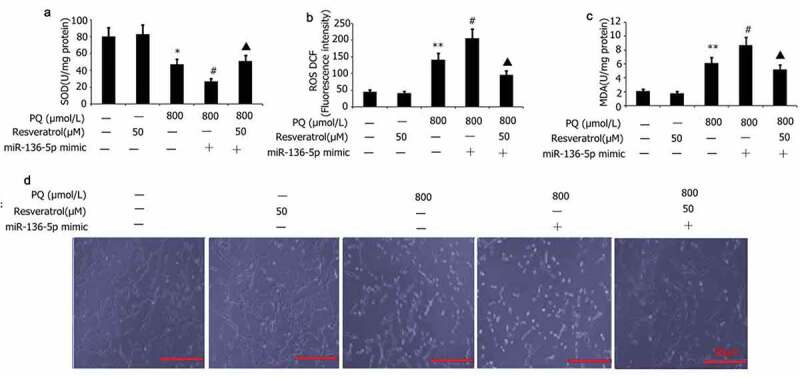
Effect of resveratrol plus miR-136-5p mimic on the oxidative stress and morphology of PQ-induced PC12 cells. Transfection of PC12 cells using miR-136-5p mimic was carried out for 24 h in the presence of PQ (800 umol/L) and resveratrol (50 uM). SOD activity and levels of ROS and MDA were determined. PC12 cells morphology was observed by AO/EB analysis, and SOD activity and MDA and ROS levels were determined. The results are expressed as mean ± SEM. *P < 0.05, **P < 0.01 versus the control group and only resveratrol group. ^#^P < 0.05 versus the PQ group. ^▲^P < 0.05 versus the miR-136-5p mimic + PQ group.

PC12 cells were transfected with miR-136-5p mimic in the presence of PQ (800 μmol/L) and resveratrol (50 μM) for 24 h to further investigate the effect of resveratrol plus miR-136-5p mimic on the oxidative stress and morphology of PQ-induced PC12 cells. Morphology observed by AO/EB analysis, and SOD activity and MDA and ROS levels were measured. As shown in Figure 7, miR-136-5p mimic attenuated SOD activity and elevated MDA and ROS levels in PQ-induced PC12 cells (Figure 7(a–c)). By contrast, resveratrol increased miR-136-5p mimic induced SOD activity and decreased activity of ROS and MDA, decreased PC12 cell apoptosis in PQ-induced PC12 cells (Figure 7(a–c)). The cells in the miR-136-5p mimic group had aggregated, the refractive index was increased, and their bodies had atrophied and became rounded and dark in color (Figure 7(d)). After the cells in the miR-136-5p mimic group were subjected to resveratrol treatment, their morphology improved significantly, the number of adherent cells increased significantly, the number of aggregated and atrophied cells decreased significantly (Figure 7(d)), and the refractive index of cells decreased (Figure 7(d)).

### Effect of resveratrol plus miR-136-5p mimic on Ki67 expression in PQ-induced PC12 cells

**Figure 8.**
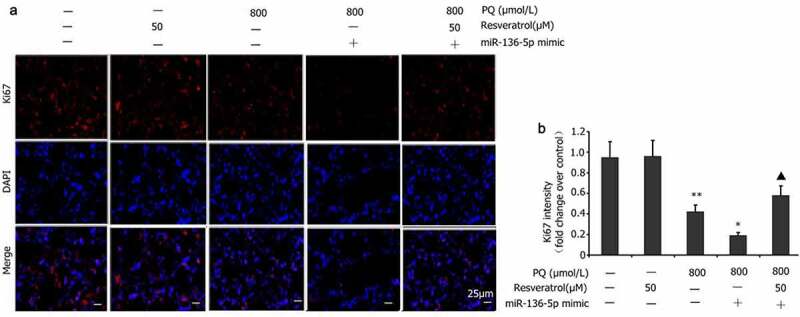
Impact of resveratrol plus miR-136-5p mimic on Ki67 expression in PQ-induced PC12 cells. In order to evaluate if miR-136-5p affected the Ki67 expression in PQ-induced PC12 cells, the cells were transfected using a miR-136-5p mimic in the presence of PQ (800 umol/L) and resveratrol (50 μM) for 24 h. Ki67 was detected by immunofluorescence, and fluorescence intensities were measured. All data are expressed as the mean ± SEM. **P < 0.01 versus the control group and only resveratrol group. ^#^P < 0.05 versus the PQ group. ^▲^P < 0.05 versus the miR-136-5p mimic + PQ group.

Ki67 expression is an important indicator reflecting the PQ-induced proliferation of PC12 cells [35]. PC12 cells were transfected with the miR-136-5p mimic in the presence of PQ (800 μmol/L) and resveratrol (50 μM) for 24 h to determine the influence of miR-136-5p on Ki67 expression in PC12 cells treated with PQ. Ki67 levels were measured via immunofluorescence. The miR-136-5p mimic reduced the level of Ki67 expression in the PC12 cells treated with PQ (Figure 8).

### Effect of resveratrol plus miR-136-5p mimic on the apoptosis, viability, and LDH activity of PC12 cells treated with PQ

**Figure 9.**
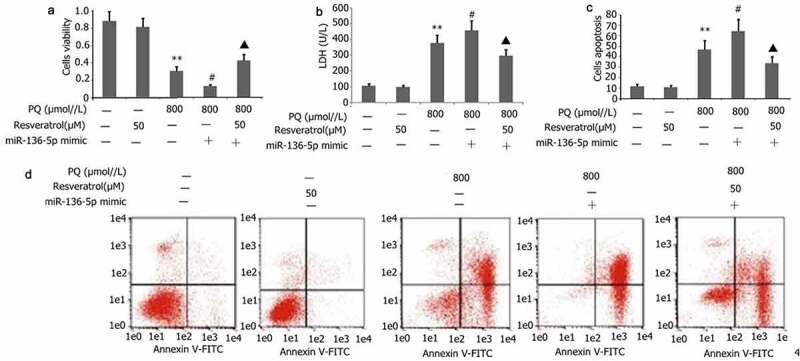
Impact of resveratrol plus miR-136-5p mimic on the viability, LDH activity, and apoptosis of PQ-induced PC12 cells. Transfection of PC12 cells with miR-136-5p mimic was performed for 24 h in the presence of PQ (800 umol/L) and resveratrol (50 μM). The PC12 cells viability was determined utilizing CCK-8, the activity of LDH was estimated according to manufacturer’s instructions, and the apoptosis of PC12 cells was measured utilizing flow cytometry. The data are expressed as mean ± SEM. *P < 0.05, **P < 0.01 versus the control group and only resveratrol group. ^#^P < 0.05 versus group. ^▲^P < 0.05 versus the miR-136-5p mimic + PQ group.

PC12 cells were transfected with miR-136-5p mimic in the presence of PQ (800 μmol/L) and resveratrol (50 μM) for 24 h to further investigate the effect of resveratrol plus miR-136-5p mimic on the apoptosis, viability, and LDH activity of PC12 cells treated with PQ. CCK-8 and flow cytometry were utilized to measure cell viability and apoptosis, respectively. As depicted in Figure 9, miR-136-5p mimic attenuated the viability but increased the apoptosis and LDH activity of PC12 cells treated with PQ (Figure 9(a–d)). Meanwhile, resveratrol reversed these effects (Figure 9(a–d)).

### Correlation between HMOX1 and miR-136-5p

Linear correlation coefficient was calculated to analyze the relationship between HMOX1 protein and mRNA expression and miR-136-5p expression in PQ-induced PC12 cells. A negative association was found between miR-136-5p and HMOX1 protein (r = −0.471, p < 0.05) and between miR-136-5p and HMOX1 (r = −0.386, p < 0.05) in the PC12 cells treated with PQ.

## Discussion

PC12 cells are a single cell strain isolated from rat adrenal chromaffin cells. Similar to nerve cells, PC12 cells are derived from the neural crest in genetics, have the same structure and function, and are relatively easy to cultivate compared with neurons [36,37]. As an in vitro cell model, PC12 cells are widely used in neuropharmacology, neuronal differentiation and development, nerve regeneration, and neurological diseases. Endogenous ROS is a normal part of the body’s aerobic respiration and is mainly produced by mitochondria, including O_2_, -OH, and H_2_O_2_. The body has an antioxidant system against ROS, including SOD, catalase (CAT), and GPx. Under normal circumstances, intracellular oxidation and antioxidants are in a dynamic balance. When the intracellular increase exceeds the antioxidant level, oxidative stress causes damage [38]. In this study, PQ increased the ROS production, oxidative stress response, and apoptosis but reduced the activity and proliferation of PC12 cells. In addition, nerve cell damage was aggravated. Therefore, PQ has neurotoxicity, and this finding is in harmony with previous works [1]. *Bacopa monnieri* supplements can reduce PQ-induced oxidative stress in mice and improve PQ-induced brain damage [39]. In the present study, treatment with resveratrol decreased the oxidative stress response and ROS production in PC12 cells and elevated the activity of antioxidant enzymes GPx and SOD. Its therapeutic effect is consistent with *B. monnieri* supplements. Oxidative stress can easily induce neuronal apoptosis in various environments [40]. Apoptosis is programmed cell death regulated by Bax, Bcl-2, caspase-3, and caspase-8 [41]. Caspase-3 activates related signal pathways by cleaving downstream effector molecules, thus leading to apoptosis. As anti-apoptotic genes, Bcl-2/Bax can be used as the upstream regulatory genes of caspase-3 and caspase-8. Bax overexpression can activate downstream caspase-3 and caspase-8 proteases and mediate cell survival or death, and Bcl-2 can form a dimer with Bax to inhibit its activity [42,43]. After the PC12 cells were cultured for 24 h with PQ, a decrease in Bcl-2 and an increase in oxidative stress, apoptosis, and Bax, caspase-8, and caspase-3 expression were observed.

HMOX1, which is affiliated to the intracellular phase II enzyme family, helps maintain cellular redox homeostasis by inhibiting ROS production and performing a critical function in oxidative stress [20]. Nrf2 has been recognized as an upstream transcription factor that modulates the activity of phase II enzymes and is inactivated under normal physiological conditions because it binds to the Kelch-like ECH-associated protein-1 (Keap1). Once the body becomes activated by ROS, Nrf2 and Keap1 are decomposed into the nucleus and combine with the antioxidant response element (ARE) to trigger the transcription of HMOX1 target gene [44]. HMOX1 is known as the initial and rate-limiting enzyme in the decomposition of heme. This enzyme catalyzes the synthesis of free ions, carbon monoxide, and hemoglobin, removes oxygen free radicals, and is rapidly induced by oxidants (such as H_2_O_2_). Its enhanced expression under oxidative stress and inflammatory conditions is crucial for cell protection [20]. The Nrf2/HMOX1 signaling pathway increases the production of antioxidant enzymes, scavenges free radicals, reduces intracellular oxidative stress, and minimizes cell degeneration. Nrf2 can protect tissue cells from oxidative damage by inducing several ROS detoxification enzymes [45]. HMOX1 is the main modulator of ROS scavenging enzymes such as GSH and CAT and inhibitsHO-1 expression and oxidative stress [45]. In this study, PQ inhibited the expression of HMOX1 and Nrf2 and the activity of the Nrf2/HMOX1 signaling pathway, leading to increased ROS production, decreased cell activity and proliferation, and increased cell apoptosis. However, resveratrol reversed these effects.

Ji et al. [46] proved that high miR-136-5p levels suppress the proliferation of mesenchymal stem cells and stimulate the apoptosis of mesenchymal stem cells by targeting BCL2. Zhang et al. [47] reported that miR-136-5p improves the growth of keratinocytes through targeted PPP2R2A regulation and thus could be used as a new therapeutic target for improving skin wound healing. The up-modulation of abnormal miR-136-5p in atherosclerosis promotes the proliferation of abnormal vascular smooth muscle cells via the ERK1/2 signaling pathway by targeting PPP2R2A. By inhibiting the production of zinc finger protein A20, miR-136-5p enhances p-NF-κB expression and thereby induce astrocytes to secrete chemokines and inflammatory factors [48]. *In vivo* experiments in mice with spinal cord injury showed that the elevated miR-136-5p expression inhibits the expression of A20 protein and increases inflammation, cell infiltration, spinal cord injury, the expression of p-NF-κB, and the secretion of pro-inflammatory factors and chemokines [48]. In the present study, PQ enhanced the expression of miR-136-5p, lowered the activity of the Nrf2/HMOX1 signaling pathway, and increased the oxidative stress response and apoptosis of PC12 cells. However, resveratrol reversed all these effects.

In this study, miR-136-5p and HMOX1 regulated PQ-induced oxidative stress in PC12 cells. TargetScan database was utilized to forecast whetherHMOX1 is the regulatory target of miR-136-5pto clarify the mutual regulation association between miR-136-5p and HMOX1. The miR-136-5p expression in PC12 cells was increased by miR-136-5p mimic and inhibitor. HMOX1 protein and mRNA expression increased, and miR-136-5p was down-regulated. Dual-luciferase reporter gene detection experiment indicated that HMOX1 is the regulatory target of miR-136-5p, which regulates the PQ-induced oxidative stress and apoptosis inPC12 cells by controlling the mRNA and protein expression of HMOX1. Further analysis revealed that miR-136-5p was negatively correlated with HMOX1 protein and mRNA expression.

Resveratrol up-regulates the expression of antioxidant enzymes, including SOD, glutathione peroxidase, and catalase by activating SIRT1 or Nrf2, down-regulating the expression of xanthine oxidase, reducing nicotinamide adenine dinucleotide oxidase and other oxidases, and inhibiting ROS production. As a result, the cardiovascular endothelial cell damage induced by oxidative stress is further reduced [49]. Resveratrol can also increase the expression of the reduced form of Parkinson protein [7], activate the PI3K/Akt/glycogen synthase kinase 3β signaling pathway, elevate the levels of glutathione and SOD, and reduce the level of inducible nitric oxide synthase, thereby inhibiting ROS generation and reducing cerebral ischemia–reperfusion injury [50]. In addition, the expression of oxidases such as xanthine oxidase is down-regulated, and nicotinamide adenine dinucleotide oxidase is reduced to inhibit ROS production and reduce cardiovascular endothelial cell damage induced by oxidative stress [51]. Ki67 is an important indicator for evaluating cell proliferation [52]. LDH is a metalloprotein that is mainly distributed in the kidney, liver, and brain; participates in glycolysis; and promotes the conversion of pyruvate into lactic acid, which is closely related to tissue cell damage. In this research, resveratrol decreased the activity of LDH [53–55] and increased the levels of Ki67 expression in PC12 cells treated by PQ. These results suggest that resveratrol can reduce PQ-induced oxidative stress and LDH activity and improve PQ-induced nerve damage.

## Conclusion

**Figure 10.**
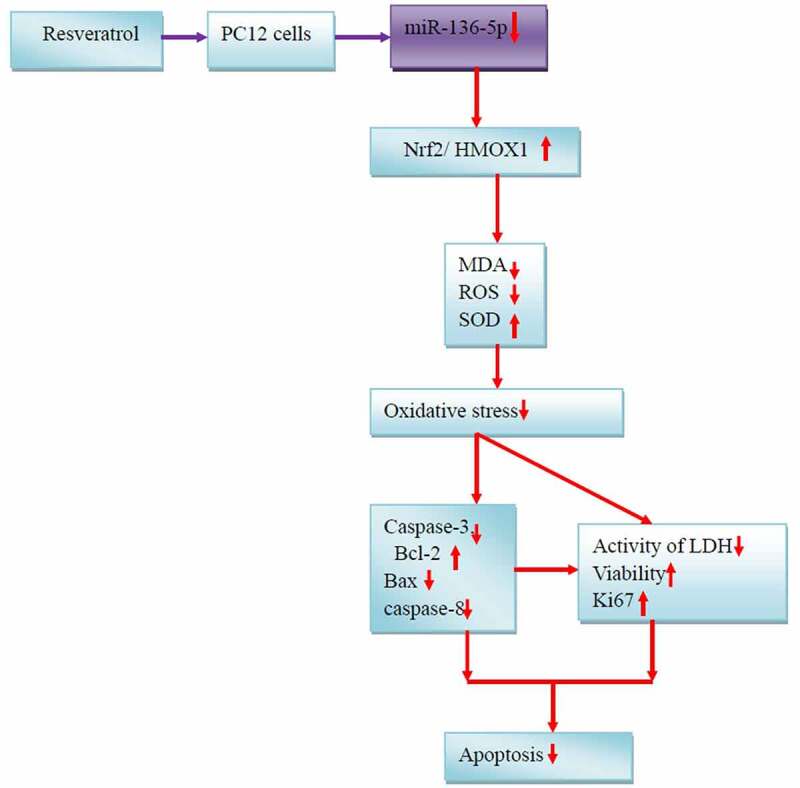
Mechanisms underlying the effects of resveratrol on PQ-induced PC12 cells.

PQ increases the miR-136-5p expression and oxidative stress of PC12 cells, inhibits the activity of the Nrf2/HMOX1 signaling pathway, and promotes the cell apoptosis. Resveratrol reduces miR-136-5p expression, oxidative stress response, and apoptosis and promotes Nrf2/HMOX1 signaling pathway activity, cell activity, and survival rate inPC12 cells treated with PQ (Figure 10). Moreover, HMOX1 was found to be the regulatory target of miR-136-5p. This study contributes to the understanding of the functional mechanism of resveratrol treatment for PQ-induced brain injury. The results promote the development of novel therapeutics and provide new gene targets for future studies. This work also provides an experimental cell basis for the resveratrol treatment of PQ-induced brain injury in humans and animals.

## Limitation

This research clarified the neuroprotective effect of resveratrol against PQ-induced PC12 cells via HMOX1 upregulation and miR-136-5p down-regulation. However, several limitations exist in this study. First, the effect of miR-136-5p on HMOX1 expression in PC12 cells may not be limited to apoptosis and oxidative stress-related proteins and pathways. Inflammation, autophagy, pyroptosis, and other proteins and pathways might also be involved. Hence, the mechanisms maybe diverse and multifaceted. Second, in addition to the Nrf2/HMOX1 pathway, many other proteins or pathways may be associated in the protective effects of resveratrol against PQ-induced PC12 cells [57]. No further research was performed on the 50% effective dose, minimum inhibitory concentration and 50% inhibitory dose of resveratrol in PC12 cells treated with PQ. The protective effect of resveratrol might involve inflammation, autophagy, pyroptosis, iron death, and other processes. Further exploration must be conducted to determine the underlying mechanisms. PC12 cells have their limitations. Any result obtained with this tumor cell line does not assure the same findings in in vivo models [56]. The addition of an in vivo animal model would be valuable. In addition, the other target genes of miRNA were not predicted in this study.

## Data Availability

The datasets utilized and/or studied in this research are accessible upon valid request.

## Highlights

- Resveratrol is a flavonoid with an antioxidant effect.
- Resveratrol can reduce the oxidative stress and apoptosis induced by paraquat (PQ).
- MiR-136-5p reduces the PQ-induced oxidative stress in PC12 cells by upregulating HMOX1 expression.
- Resveratrol reduces PQ-induced oxidative stress in PC12 cells by downregulating miR-136-5p expression.
